# “Tired but wired” - First-time parents lived experiences of sleep loss, tiredness and wellbeing during the first six months postpartum

**DOI:** 10.1371/journal.pone.0347558

**Published:** 2026-10-27

**Authors:** Shannon Hughes Spence, Aileen Leech, Hannah Goss, Siobhán O’Connor, Sinéad Smyth, Anna Donnla O’Hagan

**Affiliations:** 1 School of Health and Human Performance, Dublin City University, Dublin, Ireland; 2 School of Psychology, Dublin City University, Dublin, Ireland; Group for Technical Assistance / Asian College for Advance Studies, Purbanchal University, NEPAL

## Abstract

**Objectives:**

Sleep loss is a common experience amongst first-time parents and can result in postpartum fatigue. There is a lack of research on first-time parents’ lived experiences of sleep and sleep loss, and its associated effects on mental health, parental relationships, and overall wellbeing as new parents. We examined first-time parents’ perceptions, experiences and attitudes to sleep loss, tiredness and wellbeing during the first six months postpartum in Ireland.

**Methods:**

One-to-one semi-structured interviews were conducted with fourteen first-time parents (female n = 10, male n = 4) from October to December 2025. The interviews took place online. Data were analysed using reflexive thematic analysis.

**Findings:**

Four themes were identified: 1) the gendered experience of sleep as a new parent, 2) (in)ability to sleep, 3) postpartum wellbeing struggles and 4) resilience and adapting. These themes highlighted how first-time parents navigate their sleep in their new role as a mother or father, their difficulty getting adequate sleep, complex wellbeing struggles as a result of sleep loss, and the discovery of new ways to function when dealing with sleep loss in the postpartum period.

**Conclusions:**

These findings highlight the need for healthcare professionals to recognise postpartum sleep loss as both a physiological and psychological experience, and to incorporate holistic, routine discussions of sleep into postnatal care.

## Introduction

Sleepiness and fatigue are often used interchangeably within discussions of postpartum sleep loss, despite being two separate phenomena [1]. Sleepiness can be understood as a normal, temporary state of weariness that is often relieved by rest or sleep [1]. Although sleepiness can often improve in the later stages of the postpartum period, pervasive sleepiness can result in fatigue, a persistent lack of energy that is not always relieved by rest or sleep [2]. These two phenomena are often combined together in the general lay term of ‘tiredness’ [1]. For the purpose of this study, ‘tiredness’ is applied except in instances of reviewing previous literature. This decision was made to reflect the qualitative nature of the study, and the terminology used by the participants who did not differentiate between the two concepts.

Although sleep loss is common during the postpartum period, its prevalence varies within the literature depending on measurement methods and the amount of time since birth. One study suggests 60.4% of mothers were fatigued within the first twelve months postpartum [3]. However, most fatigue measures are not designed specifically for postpartum fatigue and as a result may not measure the true effects of this phenomenon [4]. Postpartum sleep loss has been associated with depression [5], anxiety [6], lower breastfeeding rates [7], and less effective parenting [8,9].

Several factors are associated with maternal sleep loss and fatigue in the first six months postpartum [10]. This includes caring for a newborn, physical changes in a woman’s body [11], a shift in identity, adapting to infant feeding routines [12], and domestic work [13]. Additional factors that impact maternal tiredness include financial stress, inadequate nutrition, employment, marital status and infant feeding type [10].

The role of the father has changed within the last century, with fathers being increasingly involved in their child’s care and upbringing [14]. Many fathers take paternity leave which assists them in engaging in a caregiving role and spending time with their families. Paternity leave is associated with better mental wellbeing for high-income fathers [15]. However, paternity leave was found to have minimal benefits for the mental wellbeing of fathers on a low-income, or for that of mothers [15]. Upon returning to work, men are faced with the challenge of balancing their new role as a father with their role as a worker. This can pose risks to not only their health, but to their employment stability as they are expected to be bodily and mentally present at work while experiencing sleep loss [16]. While it appears that fathers may have more sleep than mothers postpartum, tiredness also has a significant impact on men [17]. Despite this, there is a lack of research that explores how tiredness shapes the lived experience of first-time fathers.

Although maternal sleep loss and tiredness has been widely documented within the literature, much of the existing research is quantitative in nature and limits insights into how first-time mothers experience and negotiate tiredness within the gendered dynamics of early parenthood. In addition, fathers remain underrepresented in postpartum sleep research, particularly in relation to how tiredness shapes their wellbeing and perception of their paternal role [18]. As a result, there is a lack of in-depth, qualitative understanding of how both mothers and fathers experience and make sense of postpartum sleep loss and tiredness, and how these experiences can inform supportive care practices by health professionals. Therefore, this study addresses this gap by exploring first-time parents’ experiences and perceptions of sleep loss, tiredness and wellbeing during the postpartum period in Ireland. The Irish context is particularly relevant given the short paternity leave provision and gendered caregiving norms that may shape how sleep loss and early parenting are negotiated. By foregrounding such experiences, this research offers insights that can inform more holistic and inclusive approaches in postnatal support for those working with new parents.

## Materials and methods

### Design

This study employed a qualitative research design using semi-structured one-to-one interviews with first-time parents to explore their experiences and perceptions of sleep loss, tiredness and wellbeing during the postpartum period. One-to-one interviews were appropriate as they allow for an understanding of unique and personal experiences, rather than group norms or patterns [19,20]. This study was underpinned by a constructionist epistemology, recognising that experiences of sleep loss and wellbeing are socially shaped and interpreted within broader cultural and gendered contexts. This study is reported in line with the Standards for Reporting Qualitative Research [21].

### Participants

Fourteen first-time parents living in Ireland (10 = female, 4 = male) were recruited. Purposive and snowball sampling was used. Participants were recruited via social media and personal networks. Specifically, a recruitment poster was issued to maternity, family and parenting organisations who advertised the study through their email lists, social media and other networks. The inclusion criteria consisted of first-time parents over eighteen years of age with sufficient English language competencies, who had a singleton pregnancy, experienced an uncomplicated vaginal birth or planned caesarean section, and had no postnatal complications. The exclusion criteria consisted of those under the age of eighteen, who did not have sufficient English language competencies, and experienced a complicated vaginal birth or emergency caesarean section.

### Procedures

Ethical approval was obtained by Dublin City University Research Ethics Committee (DCUREC/2025/172). The semi-structured interview guide (S1 File) was developed based on protocols of other studies on postpartum sleep [22], and focused on how participants’ experiences and perceptions towards sleep have changed since having a child. A pilot interview was conducted with a first-time mother, following which, no changes were made. An information leaflet was shared and a brief demographic questionnaire was completed by participants. Written and verbal informed consent was obtained before the interviews commenced.

Data collection took place from the 29th of October 2025 to the 19th of December 2025. All interviews took place online over Zoom [23] for participant convenience. They were audio recorded via Zoom and transcribed verbatim. Any identifiable data were de-identified. Each participant was assigned a pseudonym to preserve confidentiality. Interviews lasted an average of 33.7 ± 7.5 minutes (range 26–48). The decision to cease recruitment was informed by the developing analysis, with similar patterns being observed across the participants’ accounts. Rather than seeking data saturation, the study prioritised a depth of rich data to address the research aims.

### Data analysis, reflexivity and trustworthiness

All interviews were conducted and analysed by the lead author, a PhD candidate with a background in sociology and qualitative research. She had no prior relationship with participants and adopted a reflexive approach throughout the data collection and analysis.

The data were analysed using reflexive thematic analysis (RTA) [24,25], supported by the NVivo 12 software. The six phases of RTAs were employed; familiarisation, coding, theme development, review, definitions and reporting. SHS repeatedly read the interviews after transcribing them verbatim. She noted initial codes and areas of interests before generating codes related to first-time parents’ experiences of sleep loss, tiredness and wellbeing. These codes were then collated into themes and presented to the research team (AL, ADOH, HG). Using a critical friend approach, these themes were challenged and discussed to enhance the trustworthiness of the interpretation, provided a trail of decisions that were made, and highlighted participants’ voices [26]. Descriptive statistics were used to examine the demographic information that was collected, with ages being reported with a mean and standard deviation.

## Findings

The mean age of mothers was 33.8 ± 2.1 years old, while the mean age of fathers was 35.7 ± 5.7. Table 1 outlines the participants’ names, age, feeding type, infant age and relationship to one another to contextualise their experiences that are presented throughout the findings section. Thirteen participants (or their partners) gave birth vaginally, with one participant having had an elective caesarean. Half (n = 7) of participants exclusively breastfed while the remaining half fed their infant via exclusive bottle feeding, combination feeding with breast milk or combination feeding with formula. At the time of the interviews, all mothers were on maternity leave while all fathers were working full-time. There were three couples within the sample group, resulting in less infants than parents (infants n = 11).

**Table 1:** Participant demographics table.

Participant pseudonym	Participant age	Feeding type	Age of infant in weeks (at time of interview)	Married to
Tina	34	Breast-fed exclusively	10	
Maggie	36	Breast-fed exclusively	11	
Sarah	30	Breast-fed exclusively	6	Married to Paul
Laura	31	Combination feeding with formula	10	
Mark	41	Bottle-fed exclusively	4	
Sabrina	32	Bottle-fed exclusively	27	Married to Max
Nina	35	Breast-fed exclusively	19	
Shauna	34	Bottle-fed exclusively	25	
Paul	29	Breast-fed exclusively	10	Married to Sarah
Max	34	Bottle-fed exclusively	30	Married to Sabrina
Nicole	37	Breast-fed exclusively	7	
Molly	33	Breast-fed exclusively	2	
Ruth	35	Combination-feeding with breast milk	24	Married to Jack
Jack	39	Combination-feeding with breast milk	24	Married to Ruth

The findings are presented under four primary themes: 1) the gendered experience of sleep as a new parent, 2) (in)ability to sleep, 3) postpartum wellbeing struggles and 4) resilience and adapting. See S2 File for additional participant quotes to support each theme and subtheme.

### 1. The gendered experience of sleep as a new parent

This theme showcased how participants navigated sleep in their new role as a first-time mother or father. This primary theme consisted of three subthemes: Paternity leave; Mother’s labour after the labour; Navigating the role of fatherhood.

#### 1.1. Paternity leave.

First-time parents’ experiences of sleep were distinctively gendered. One such factor was paternity leave, a two week period that new fathers are entitled to take off work within the first six months after a baby is born. This study found that paternity leave shapes the sleep experiences of both parents. Extended paternity leave beyond Ireland’s statutory two-week provision was associated with relief and gratitude amongst first-time fathers, and sympathy for those confined to the minimum entitlement.

*A big help to me was I took just over three months of paternity leave. All paid, thankfully… I just couldn’t understand other new dads who take two weeks and then they come back, and they’re still in that early stage of two to three hour intervals of feeding… I was still able to get pretty consistent blocks of four or five hours sleep, and so the return to work wasn’t too tough for myself.* (Max)

Mothers also reported the benefit of extended paternity leave, having felt a greater sense of support from their partners. The fathers presence and ability to care for the child was essential to the mother’s own wellbeing when paternity leave was greater than the statutory minimum. *“I’m really blessed that my husband has six weeks paternity leave*” (Molly). With only the statutory minimum of paternity leave, returning to full-time work while sleep-deprived from frequent infant waking was described as “*jolting*” (Mark).

#### 1.2. Mother’s labour after the labour.

Mothers’ labour after birth refers to the domestic and emotional labour she performs due to gendered social norms, which further contributes to tiredness. This labour is often normalised and expected due to gendered social norms such as “*when you’re a mom with a newborn, most of* *the things are on the mom*” (Maggie). Five of the female participants expressed their frustration at the advice ‘sleep when the baby sleeps,’ commonly offered by family and friends. They felt unable to nap during the day to mitigate tiredness and instead had to attend to domestic chores.

*Some people have said ’forget about the laundry, forget about the cooking, forget about the cleaning, let everything sit and go to sleep when the baby is asleep’. But that’s not going to help me. I can’t just not eat and not have clean clothes… that irritates me*. (Sarah)

Additionally, mothers’ engage in maternal labour through breastfeeding, with night-time feeding being their sole responsibility. Tina shared that she was exclusively breastfeeding “*so all of the wake-ups are [hers]*”. However, although breastfeeding mothers wake during the night, a fathers sleep is still disturbed, which can become an issue when he returns to work.

*My partner is breastfeeding so there’s no point in me having to wake up as well. But because we sleep in the same room, I do wake up when she [the baby] starts crying.* (Paul)

#### 1.3. Navigating the role of fatherhood.

When gendered expectations positioned women as responsibile for domestic labour and night feedings, fathers expressed uncertainty about their role within the home. This subtheme focuses on that uncertainty. This uncertainty, alongside feelings of guilt related to gendered caregiving exacerbated fathers’ tiredness. Instead of resting, fathers described a preference for staying busy to ensure they are available to assist with the infant if needed.

*When [wife] is with the baby, there’s no point in two of you sitting there with the baby... Rather than me just sitting there doing nothing, I try to keep myself occupied. But if I go to bed, you probably feel like you’re not pulling your weight.* (Jack)

Being able to support the mother as part of the fathers role was articulated regarding the ability to share duties around bottle-feeding, with Max sharing he was “*really gratefu*l” to support his wife when “*she needs to have a shower, or put the wash on*”. Participants also reported deliberate team working strategies.

*Myself and my husband decided we’d split the night in two. I would sleep from 8 pm until 2am. And then basically from 2am until 8am, it was my job then to care for [daughter] if she woke. And that massively helped me.* (Sabrina)

### 2. (In)ability to sleep

This theme explored participants’ difficulty getting adequate sleep. It consisted of two subthemes: Broken sleep: quality vs quantity and Survivalist sleep hygiene and strategies.

#### 2.1. Broken sleep: perceived quality vs quantity.

Although all participants experienced interrupted sleep after becoming parents, an important differentiation was in their perceptions of sleep quality. While some parents could function on a certain number of hours of uninterrupted sleep and feel rested, others could not. When sleep in between the disruption was experienced as restorative, participants described more positive sleep experiences.

*I don’t think it [her sleep] has changed a lot, it has just changed that it’s completely broken. I sleep 2 hours here, then I wake up. I sleep with an eye open, things like that. It’s obviously not as deep as it used to be. And it’s probably a really light sleep. But my awareness is that I wake up and I’m rested, so, it’s not that bad*. (Maggie)

In contrast, others felt that although the duration of their sleep was significant, the frequent waking led them to perceive their quality of sleep as poor.

*Even though I know I’m getting 7 hours [sleep], which should be enough to function on, when it’s broken, it is a lot more difficult. So, I think that broken sleep is a lot more challenging. It doesn’t feel like I’ve got 7 hours.* (Laura)

Perceptions of restorative sleep are important and individual framing may play a role in that. Paul decided to manage his experience of tiredness by acknowledging he was going to “*be quite tired and fatigued*” and treat this postpartum period the same as previous times in his life where he did not “*sleep well*”. By positioning his perspective of tiredness in this manner, he was able to recall previous experiences that he was able to overcome and approach his tiredness in a similar way.

#### 2.2. Survivalist sleep hygiene and strategies.

Participants employed several sleep strategies to cope with tiredness and sleep loss. All participants found comfort in both evidence-based and non-evidence based sleep hygiene practices, with each participant mentioning at least one strategy they tried to help with their sleep loss. This included taking magnesium supplements, drinking chamomile tea, using eye masks, mouth tape, white noise machines, minimal screen time, red lights and blackout blinds. Good sleep hygiene was generally seen as beneficial to improve sleep quality and reducing tiredness where possible. There was a tension between knowledge and practice when it comes to screen time at night for first-time parents. Paul shared he has “*a habit of reading the news on [his] phone as [he] gets into bed, which [he] shouldn’t do*.” The role of good sleep hygiene practices was viewed as complex because there were times that new parents felt they needed to remain alert at night. Nicole for example reported using screen time to stay awake to safely care for her child although she recognised it may disrupt her sleep later.

*I know I shouldn’t be using it (her phone) overnight, but it’s just purely to stay awake so I don’t fall asleep. I’m worried about that and I don’t want to drift off when she’s feeding*. (Nicole)

### 3. Postpartum wellbeing struggles

This theme highlighted the complex wellbeing struggles participants experienced as a result of their tiredness. It consists of two subthemes: “Tired but wired” and Anxiety and sleep.

#### 3.1. “Tired but wired”.

Participants expressed feelings of tiredness and a strong desire for sleep. Yet, when their baby was asleep at night, they struggled to switch off due to worry and intrusive thoughts about the baby’s safety. Fears included the baby being too hot or cold, falling asleep while holding the baby, or, as Sabrina shared “*every two seconds*” she would panic and think “*she (the baby) is not breathing*”. These worries prevented parents from sleeping, despite their tiredness.

*I saw this thing online, ‘tired but wired’, and it’s that you’re so anxious that your nervous system is like that. Even though (daughter) might sleep through the night, I could still be up three times checking on her and worrying about her.* (Ruth)

#### 3.2. Anxiety and sleep vs anxiety about sleep.

Eight of the participants mentioned how their anxiety and sleep had been intertwined, while also highlighting their anxiety about sleep. From feeling overwhelmed to being referred to a perinatal mental health team, eight of the participants named a bidirectional relationship between sleep and mental health during the postpartum period. Additionally, a lack of sleep combined with mental health struggles affected bonding with infants.

*I was linked in with the perinatal mental health team for my anxiety. I physically couldn’t sleep. I was getting to the stage where I was going to need to actually be tranquilized. I remember speaking to the (mental health team), panicking about how little sleep I was getting and how I was finding it really hard to function, and it was massively affecting my bonding with [daughter].* (Sabrina)

### 4. Resilience and adapting

This theme highlights how participants found new ways to function when experiencing tiredness during the postpartum period. It consists of four subthemes: Expecting the worst; Prioritising the baby’s sleep; “It will pass”; A new way of functioning.

#### 4.1. Expecting the worst.

Participants shared that they had anticipated severe tiredness before becoming parents, with five sharing that they felt unprepared when faced with their actual levels of sleep loss that they experienced. This sleep loss heightened their worries and anxiety during the postpartum period. Sabrina shared it was one of her “*biggest fears having a baby*” due to her association with poor sleep and mental health struggles. However, anticipating severe tiredness also meant that participants were pleasantly surprised when their experience was less challenging than expected.

*My expectation was that it was going to be horrible. That we weren’t going to sleep at all the entire night. So because I had prepared myself so badly, now, when it comes to the nighttime, my sleeping is completely broken through the night, but in between, I sleep and I wake up and I’m rested.* (Maggie)

#### 4.2. Prioritising the baby’s sleep.

Parents reported prioritising their baby’s sleep over their own. Efforts were focused on improving the infant’s sleep rather than supporting their own.

*I’m more just focused on her sleep rather than my own. I’m saying ‘oh, she got 3 hours sleep’. That never means that I’ve gotten the same amount of sleep, because it takes a while for me to fall asleep after she does*. (Sarah)

However, as the infants grew, parents began to recognise the importance of prioritising their own sleep.

*I don’t think I realised until maybe about 3 months in how I needed to prioritise it, because your brain… my brain was wired to her first, you know?* (Sabrina)

#### 4.3. “It will pass”.

Participants coped by framing tiredness as a temporary phase, drawing comfort from the knowledge that others had faced similar experiences.

*You’re going to have a lack of sleep, but you do realize that realistically there’s an ending to this. My mother, father, aunts and uncles, more experienced people have seen it all, or went through this. It’ll pass…So you have to put your trust in them.* (Mark)

Participants reported frustration when professionals offered the same reassurances, emphasising the limited support available for first-time parents managing tiredness. Being tired and fatigued was seen as an inevitable part of parenthood and dismissed.

*I mentioned it to the GP. I just said ‘I have to sleep, I’m going to go insane’. Everybody said to me ‘it’ll pass, it’ll pass’. I feel when you go to healthcare professionals, there’s this perception that that’s just part of it, and it will pass.* (Molly)

#### 4.4. A new way of functioning.

Four participants reported that they were surprised they were able to adapt and function on less sleep after becoming parents. Before parenthood, they would require seven to eight hours of sleep, whereas now they can function on much less.

*Before we had [daughter], if I didn’t get a full night’s sleep, I’d be in a shocking mood or it would have a big impact the following day. But, since I’ve had my baby, I’ve been surprised at just kind of how quickly you adapt to operating on less sleep.* (Max)

## Discussion

The aim of this study was to examine first-time parents’ experiences and perceptions of sleep loss, tiredness and wellbeing during the postpartum period. Four key themes were identified through thematic analysis of semi-structured interview data; how gender shapes sleep experiences, the psychological element of sleep loss, wellbeing challenges, and the resilience of first-time parents.

This study increases the understanding of first-time parents’ experiences of sleep loss by considering gendered dynamics. Between 2019 and 2022, at least 47% of fathers in Ireland were not availing of their entitled two weeks paid paternity leave following the birth of their child [27]. Based on these findings it would seem reasonable to suggest that at two weeks post child birth (and oftentimes earlier), mothers in Ireland are assuming the majority of responsibilities of caring for their newborn. Although the role of the father has changed within the last century [16], it is clear that beyond and even within the first two weeks postpartum, more responsibility typically falls on mothers than fathers [28,29]. The gendered dynamics of parenthood must be considered by healthcare professionals who work with this cohort to highlight the importance of shared responsibility, as literature suggests coparenting relationship quality is lower amongst couples where women engaged in more housework and childcare than wanted [29], potentially impacting both sleep and wellbeing.

However, the structural policies that shape this unequitable distribution of labour must also be acknowledged. Ireland’s state paid paternity leave is the minimum requirement under European Union laws and legislation [30]. The state payment for paternity leave is currently €299 per week, with economic loss being a possible factor for the low uptake of paternity leave. Internationally, the average paid paternity leave amongst members of the Organisation for Economic Co-operation and Development (OECD) is 2.4 weeks [31], with Spain offering the longest period of paid paternity leave at 16 weeks. However, amongst other OECD countries, fathers can avail of paid leave for 8.1weeks while retaining their full earnings, with Japan offering 31.1 weeks at full earning [31]. The Irish state’s lack of financial support for new fathers reinforces a culture where women in Ireland still carry out the majority of care during the postpartum period, while fathers miss out on the early months of parenthood due to financial necessity. In line with other countries, Irish policy should consider extending the duration and payment of paid paternity leave beyond the minimum statutory requirements. Evidence suggests that well-compensated paternity policies are associated with higher uptake amongst new fathers and greater gender equality [31], and potentially, increased opportunities for rest and wellbeing during the postpartum period.

Previous studies which examined the prevalence of postpartum sleep loss highlighted several factors that contribute to postpartum tiredness, including frequent nighttime infant care, physical changes in the body and environmental triggers such as poor sleep hygiene practices [11,12]. The findings of our study extend the research on postpartum sleep loss by identifying that sleep loss is not only physiological, but psychological for first-time parents. Even when an infant is sleeping and despite parental tiredness, first-time parents may struggle to sleep due to their anxiety surrounding the wellbeing of their infant. This supports wider literature that highlights heightened anxiety and hypervigilance as being common amongst mothers within the first eight weeks postpartum [32]. Future research, interventions, and healthcare workers who address parental sleep loss must consider the psychological barriers to sleep and can relieve such concerns by providing first-time parents with information on common sleeping patterns and behaviours of infants within the first six months postpartum [33].

Our findings demonstrate that despite the prevalence and difficulty of postpartum sleep loss, first-time parents find new ways to function and cope. There is however a risk of relying on individual resilience to adapt to sleep loss. Informal support from family and friends can be an important factor in preventing depressive symptoms in the postpartum period [34]. However, social support has been found to decline postpartum, increasing the risk of maternal mental health difficulties [34]. Where social support is unavailable, this research recommends engaging in more ‘formal’ supports, which for participants in this study, was not widely available. For example, healthcare workers can support first-time parents’ sleep by guiding them towards evidence-based sleep hygiene practices and an awareness of existing available support [35]. To be effective and widely reached, these supports need to be easily accessible to first-time parents. As healthcare workers are well positioned to recognise the psychological and physical dimensions of postpartum sleep loss, routine discussions of sleep should be incorporated into postnatal care. This should include addressing gendered divisions of care and offering practical strategies for managing sleep in the early postpartum period. Furthermore, future research should take into consideration the gendered nature of sleep loss when examining first-time parents’ experiences of sleep.

There were several limitations to our study. As the study aimed to explore lived experiences, generalisability may not be applicable. Of the fourteen participants, only four were fathers. Although this study captures their experiences, future research could benefit from a wider sample of first-time fathers. Secondly, all participants were a part of a couple, indicating support from a partner. The lived experience of a single/solo parent may greatly differ from the participants within this study. Although some demographic information was collected on the participants, no socio-economic information was gathered, which is known to impact the wellbeing of new parents. Lastly, as participants were recruited through maternity/parenting mailing lists and social media, the sample could likely reflect parents already interested in or engaged within parenting/wellbeing communities.

## Conclusion

Postpartum sleep loss has been associated with depression, anxiety, lower breastfeeding rates, and less effective parenting. The findings of this study extend research on postpartum sleep loss by increasing the understanding of first-time parents’ experiences of sleep loss within gendered dynamics. It also identifies that sleep loss is not only physiological, but psychological for first-time parents. Lastly, this study shows that despite the prevalence and difficulty of postpartum sleep loss, first-time parents find ways to function and cope.

Preparing healthcare professionals to engage with postpartum sleep may enhance the support provided to new parents. Antenatal and postnatal education programmes should better equip parents by setting realistic expectations around sleep loss and tiredness, and encourage shared caregiving practices and provide coping strategies that acknowledge the emotional and relation challenges that can arise during the postpartum period.

## Supporting information

S1 File Semi-structure interview schedule. (DOCX)

S2 File Additional participant quotes for key themes and sub-themes. (DOCX)
