# Supplementary material for: “Tired but wired” - First-time parents lived experiences of sleep loss, tiredness and wellbeing during the first six months postpartum
Source: PLoS One. 2026 Oct 27;21(10):e0347558. doi: 10.1371/journal.pone.0347558 (PMC13712679; doi:10.1371/journal.pone.0347558)
Supplement: Supplementary file 1 [file pone.0347558.s001.docx]

*Supplementary Material A. Semi-structure interview schedule*

*The purpose of this interview is to help us understand more about new parents’ perceptions, experiences and attitudes to sleep, fatigue, and tiredness during the postpartum period in Ireland. Firstly, I want to define some of the terms we will be discussing…Tiredness is a normal, temporary state of weariness, often relieved by rest or sleep. Fatigue, on the other hand, is a more persistent and pervasive feeling of exhaustion that may not be fully resolved by rest and can significantly impact daily life. Sleep Health refers to the overall state of your sleep (e.g., quality, duration, regularity) while Sleep Hygiene refers to the specific practices, behaviours (e.g., consistency, bedtime routine) and environmental factors (e.g., dark, cool room) that support good sleep health.*

*I’m going to ask you a few questions about your perspective on your sleep generally, and in relation to your role as a new parent. Please describe as much of your experience as possible. If there’s a question that you’d prefer not to answer or you need a break, that is no problem, and it won’t impact your relationship with DCU or any organisation that you may be associated with. Just to remind you that the answers you give me are completely confidential.*

*Before we start do you have any questions for me?*

*Are you happy for me to record this?*

*Are you happy to start the interview?*

*Can you give verbal consent as well?*

***Section 1: Introductory Questions on Sleep – Awareness, Knowledge***

***1.*** *How important is sleep to you as a new parent?*

*a.   How do you think being a new parent affects your perception of sleep?*

***2.*** *What is your experience of sleep as a new parent?*

*a.   You mentioned_______, why do you think being in the postpartum period leads to that experience?*

*b.   Has your sleep changed since having a baby? How so?*

***3.*** *What do you think is preventing you from getting as much sleep as you might like or need now?*

*a.   You mentioned_______, do you think there is anything else?*

*b.   How do you think this is affecting your life as a new parent?*

*c.   Do you feel you have control over how much sleep you get?*

***4.*** *Are there any things that you have done to improve your sleep/tiredness/fatigue during the postpartum period?*

*a.   You mentioned ____ is important, could you tell me why that is?*

***5.*** *Are you aware of any supports (formal or informal) available to help new parents struggling with their sleep and/or tiredness or fatigue and its effects during the postpartum period?*

*a.   When you say ____, who exactly do you mean?*

*b.*   *If supports regarding sleep/sleep health/sleep hygiene were available to new parents, do you think they would engage with them? Why or why not?*

***Section 2: Perceptions of Sleep***

***6.*** *Do you think it would be beneficial for new parents to learn about sleep/sleep health/sleep hygiene and how to manage their sleep during the postpartum period? Why/not?*

***7.***      *What would facilitate you in accessing supports (formal or informal) and information for sleep/sleep health/sleep hygiene during the postpartum period?*

*a.   You suggested ____ could be a facilitator, could you explain that a bit more for me?*

*b.   Just going back to when you said____, can you explain that a bit more?*

*c.   You mentioned ____, how do you think that acts as a facilitator?*

*d.   Would you be able to tell me more about how/why ____ is a facilitator?*

***8.***      *What would prevent you from accessing supports (formal or informal) and information for sleep/sleep health/sleep hygiene during the postpartum period?*

*a.   Just going back to when you said____, can you explain that a bit more?*

*b.   You mentioned ____, how do you think/perceive that would prevent someone from seeking help?*

*c.   Would you be able to tell me more about how/why ____ is a barrier?*

*d.   What are other demands that might get in the way of seeking help?*

***9.*** *If sleep/sleep health/sleep hygiene supports for the postpartum period were made available for new parents, do you think you would engage with them? Why/not?*

*a.*   *What kinds of supports (formal or informal) for sleep health/sleep hygiene would you be more likely to engage with? Why?*

***Section 3: Sleep Intervention Programme for First-Time Parents***

*You’ve brought up several different barriers and facilitators. We’re considering that information and supports around sleep for new parents be an important factor to address some of those points you have mentioned.*

***10.***          *In your opinion, as a new parent, what is the best way to deliver this type of sleep information and support programme to new parents? (i.e. online, in person, hybrid)*

*a.   When should it be done?*

*b.   How much time should it take? (i.e. one hour per week, one day, discussion group, evenings, weekends)*

*c.   Should it be completed as a group or individually?*

*d.   Who should lead the programme (if done as a group)? (i.e. psychologist, trained facilitator with a medical background, Public Health Nurse)*

*e.   Does there need to be several different programmes?*

*f.    Would you like to see equipment be used in the programme?*

*g.   What format should it take (i.e. classroom, workshop, bite size modules, day seminar)*

*h.   How often should this be done?*

***11.***          *What content needs to be the focus of the sleep education talk for new parents?*

*a.   When you say ____, who exactly do you mean?*

***12.*** *If this were to be developed, would this be something that you would engage with? Why or why not?*

*a.*   *Who needs to support the programme for it to be successful in your opinion?*

*b.*   *How can leaders or other parents impact the adoption of the programme?*

*c.*   *What role have supervision/motivation/feedback in the long-term success of the programme?*

***13.***          *We talked a bit about being able to manage sleep during the postpartum period and supports for new parents’ sleep. Is there any additional topics that you think should be included in a specialised education programme on sleep for new parents?*

*a.   How do you think education could be organised to reach a wider audience?*

*b.   Does the programme need to include anything else outside of sleep, tiredness, and fatigue?*

***Closing***

***14.***          *Is there anything else you think is important when considering ways to reach new parents and support them in as regards their sleep, tiredness, and fatigue during the postpartum period?*

***15.***          *From all the things that we have spoken about today, what is the most important factor for us to consider?*

***16.***          *Is there anything we haven’t had an opportunity to discuss which you think is important to consider?*

***Additional prompts***

*· Why do you think ____ helps with adoption?*

*· You mentioned ____ earlier, is there anything more you can say about that?*

*· Can you tell me more about how/why ____ makes it easier for the programme to be adopted? · When you say ____, who exactly do you mean?*

*· When you say ____, what do you mean by that?*

*· Would you mind explaining your thoughts on implementing ____ more for me?*

*· Just going back to when you said____, can you explain that a bit more?*

*· When you say the strategy should include ____, are you talking about ____?*

*· You brought up ____, how would you like to see this put into practice?*

*· You mentioned _____, what is the most important in your opinion?*

*· You mentioned ____, how do you think/perceive that acts as a barrier to adoption?*

*· What exactly do you mean by ___?*

*· You made a point about ____, is there anything else you can say about that?*

*· Why do you think/perceive ____ is a barrier to uptake?*

*· Would you be able to tell me more about how/why ____ is a barrier?*

*· You think ______, could you tell me more about this?*

*·You said that ____ should be part of the education, would you mind explaining this more? ·You mentioned ____ is important, could you tell me why that is?*

**Prompts will be dependent upon the ideas put forward by the participant in relation to sleep, tiredness, and fatigue experiences and/or comments.*
