# Supplementary material for: “Tired but wired” - First-time parents lived experiences of sleep loss, tiredness and wellbeing during the first six months postpartum
Source: PLoS One. 2026 Oct 27;21(10):e0347558. doi: 10.1371/journal.pone.0347558 (PMC13712679; doi:10.1371/journal.pone.0347558)
Supplement: Supplementary file 2 [file pone.0347558.s002.docx]

*Supplementary Material B. Additional participant quotes for key themes and sub-themes*

| *Theme and subtheme* | *Quotes* |
| --- | --- |
| *Theme 1:* The gendered experience of sleep as a new parent |  |
| *Subtheme:* Paternity leave | “I suppose to be honest, you get 2 or 3 weeks paternity, and then obviously, you're back into work, so that's quite a jolting thing, you know?” Mark, aged 41, 4 week old baby |
| *Subtheme:* Mother’s labour after the labour | “I know a lot of people say ‘nap when the baby naps’. I find that during the day when my baby naps… I feel like that's my time to eat, or it's my time to catch up on the dishes, or whatever it might be.” Molly, aged 33, 2 week old baby  “My partner hasn't been doing any nighttime feeds or anything like that. It's been quite challenging, I suppose, my sleep so far.” Nicole aged 37, 7 week old baby |
| *Subtheme:* Navigating the role of fatherhood | “And like, so many guys, when I talked to them, they were like, oh, well, she's   breastfeeding, so there's not a whole lot I can do.” Nina, aged 35, 19 week old baby |
| *Theme 2:* (In)Ability to sleep |  |
| *Subtheme:* Pre-birth sleep issues | “My last 5 weeks of pregnancy, I had insomnia. I just woke every night. So I suppose it was starting to kind of wax and wane before [child] was born” Molly, aged 33, 2 week old baby |
| *Subtheme:* Sleep disruption | “Before baba you could pretty consistently get a full night's sleep, and you wouldn't have to disturb yourself. Apart from, say, like, going to the bathroom or whatever else. But it's definitely more fragmented, or at least it had been, at the start, not so much now.” Max, aged 34, 30 week old baby  “I'm just kind of surviving on the…a few hours overnight, but I do stay awake, during the day.” Nicole, aged 37, 7 week old baby. |
| *Subtheme:* Sleep hygiene | “We did get blackout blinds, which I would 10 out of 10 recommend. I was using a small lamp to do things in the middle of the night, which was causing everything to be really bright, definitely was waking me up. So we actually have a red lamp that I use instead, which obviously is much more gentle on the eyes. It feels like it kind of doesn't wake me.” Tina, age 34, 10 week old baby  “We're definitely using a lot more white noise and. [wife] has an eye mask. I have the mouth tape or whatever because I snore pretty consistently.” Max, aged 34, 30 week old baby |
| *Subtheme:* Making active choices to improve sleep | “I suppose you get maybe 2 hours, or 1 hour. My wife and I split it intermittently, you know what I mean? We kind of balance trying to balance who gets up” Mark, aged 41, 4 week old baby |
| *Theme 3:* Postpartum wellbeing struggles |  |
| *Subtheme:* “Tired but wired” | “Everyone was trying to help me sleep and I couldn't do it.. If I couldn't hear her, that made me anxious. I'd hear the phantom crying, and I'd be convinced something was wrong… I used to be a pediatric nurse and it made my anxiety worse, because I kept expecting a worst-case scenario with her… I was so sleep-deprived worrying about her.”  Sabrina, aged 32, 27 week old baby |
| *Subtheme:* Mental health issues | “I really, really, really struggled with my mental health… When you're that tired, and the hormones mixed in… that is probably one of the biggest factors of falling into ruts or depression, or postnatal anything.” Shauna, aged 34, 25 week old baby |
| *Theme 4:* Resilience and adapting |  |
| *Subtheme:* “It will pass” | “And so I suppose it's just kind of accepting a lot, and not, putting so much pressure on me or her. That kind of helps the mindset of, like, relating to sleep, and knowing that it's just a phase, and knowing that it hopefully will get better, at least that's what people are telling me.” Nicole, aged 37, 7 week old baby |
| *Subtheme:* Expecting the worst | “Facing into becoming a new parent, it was the part that I was most nervous about, was how to manage on less sleep. You're thinking about all the time, in terms of how long is the baby sleeping, and then how much sleep have I got? So, yeah, it's something that's on your mind a lot more, instead of just something that happens every day.” Laura, aged 32, 10 week old baby |
| *Subtheme:* Prioritising the baby’s sleep | “My sleep is not as important as his sleep. His sleep impacts the day a lot more than mine does. It's just kind of downgraded.” Tina, age 34, 10 week old baby |
| *Subtheme:* A new way of functioning | “I think that my standards have lowered. Before, you know, I would have always thought I really want to get 7-8 hours of sleep every night, whereas now even if I can just grab a window of 1-2 hours of sleep, that is enough for me.” Molly, aged 33, 2 week old baby  “I'm still well able to get up and meet people and have a coffee and be in good form. It's not having, like, a really negative impact on me day-to-day yet. I don't know how long of this interrupted sleep will start to impact me, but at the moment, I still feel like myself.” Sarah, aged 30, 6 week old baby |
