# Supplementary material for: “Tired but wired” - First-time parents lived experiences of sleep loss, tiredness and wellbeing during the first six months postpartum
Source: PLoS One. 2026 Oct 27;21(10):e0347558. doi: 10.1371/journal.pone.0347558 (PMC13712679; doi:10.1371/journal.pone.0347558)
Supplement: Supplementary file 3 [file pone.0347558.s003.docx]

| **Editor comments** | **Response/revision** |
| --- | --- |
| Thank you for stating the following financial disclosure:  “This work was supported by the HRCI/HRB Joint Funding Scheme HRCI-HRB-2024-016"  Please state what role the funders took in the study.  If the funders had no role, please state: "The funders had no role in study design, data collection and analysis, decision to publish, or preparation of the manuscript."  If this statement is not correct you must amend it as needed.  Please include this amended Role of Funder statement in your cover letter; we will change the online submission form on your behalf. | Thank you. We have added this statement into the cover letter and online form. |
| In the online submission form you indicate that your data is not available for proprietary reasons and have provided a contact point for accessing this data. Please note that your current contact point is a co-author on this manuscript. According to our Data Policy, the contact point must not be an author on the manuscript and must be an institutional contact, ideally not an individual. Please revise your data statement to a non-author institutional point of contact, such as a data access or ethics committee, and send this to us via return email. Please also include contact information for the third party organization, and please include the full citation of where the data can be found. | Thank you. This has been updated to the DCU ethics committee contact. |
| "We note that there is identifying data in Table 1. Prior to sharing human research participant data, authors should consult with an ethics committee to ensure data are shared in accordance with participant consent and all applicable local laws.  Data sharing should never compromise participant privacy. It is therefore not appropriate to publicly share personally identifiable data on human research participants. The following are examples of data that should not be shared:   -Name, initials, physical address  -Ages more specific than whole numbers  -Internet protocol (IP) address  -Specific dates (birth dates, death dates, examination dates, etc.)  -Contact information such as phone number or email address  -Location data  -ID numbers that seem specific (long numbers, include initials, titled “Hospital ID”) rather than random (small numbers in numerical order)  Data that are not directly identifying may also be inappropriate to share, as in combination they can become identifying. For example, data collected from a small group of participants, vulnerable populations, or private groups should not be shared if they involve indirect identifiers (such as sex, ethnicity, location, etc.) that may risk the identification of study participants. | The names in the table 1 are pseudonymised and not a reflection of the participants actual names. This is in line with DCU ethical approval and was agreed with the participants that they would be given fake names in any publications. We believe this is reflects standards of confidentiality. However, if it is preferable for pseudonyms to be removed and replaced with ‘participant 1, female’ for example, we are happy to do so. |
| Thank you for revising your work.  I appreciate that you have addressed all reviewers' feedback, and I think the paper is much clearer now.  As you have justified the inclusion of Irish participants based on the short paternity leave provided, please discuss this aspect further in the discussion section. You may add a paragraph comparing and contrasting the Irish situation with other European or high-income countries regarding the duration of paternity leave and/or the distribution of leave between mother and father. This would enhance interest among international readers and improve the paper's relevance at the international level. | Thank you for the comment and suggestion for improving the manuscript. This suggestion has been implemented and inserted into the manuscript, from line 366 to 380. It reads:  “However, the structural policies that shape this unequitable distribution of labour must also be acknowledged. Ireland’s state paid paternity leave is the minimum requirement under European Union laws and legislation^30^. The state payment for paternity leave is currently €299 per week, with economic loss being a possible factor to the low uptake of paternity leave. Internationally, the average paid paternity leave amongst members of the Organisation for Economic Co-operation and Development (OECD) is 2.4 weeks,^31^ with Spain offering the longest period of paid paternity leave at 16 weeks. However, amongst OECD countries, fathers can avail of paid leave of 8.1weeks while retaining their full earnings, with Japan offering 31.1 weeks at full earning^31^. The Irish state’s lack of financial support for new fathers reinforces a culture where women in Ireland still carry out the majority of care during the postpartum period, while fathers miss out on the early months of parenthood due to financial necessity. In line with other countries, Irish policy should consider extending the duration and payment of paid paternity leave beyond the minimum statutory requirements. Evidence suggests that well-compensated paternity policies are associated with higher uptake amongst new fathers and greater gender equality^31^, and potentially, increased opportunities for rest and wellbeing during the postpartum period. “ |
| **Reviewer 1** | **Response/revision** |
| ABSTRACT  This section is well structured and informative. No comments. | Thank you for your feedback. |
| INTRODUCTION  The introduction is well structured.  However, it is not clear what the scientific gap in the literature is and how this manuscript contributes to clinical practice. | Thank you for this comment. The introduction has been amended from line 89-100 and now reads as follows: “Although maternal sleep loss and tiredness has been widely documented within the literature, much of the existing research is quantitative in nature and limits insights into how first-time mothers experience and negotiate tiredness within the gendered dynamics of early parenthood. In addition, fathers remain underrepresented in postpartum sleep research, particularly in relation to how tiredness shapes their wellbeing and perception of their paternal role.^20^ As a result, there is a lack of in-depth, qualitative understanding of how both mothers and fathers experience and make sense of postpartum sleep loss and tiredness, and how these experiences can inform supportive care practices by health professionals. Therefore, this study addresses this gap by exploring first-time parents' experiences and perceptions of sleep loss, tiredness and wellbeing during the postpartum period in Ireland. The Irish context is particularly relevant given the short paternity leave provision and gendered caregiving norms that may shape how sleep loss and early parenting are negotiated.. By foregrounding such experiences, this research offers insights that can inform more holistic and inclusive approaches in postnatal support for those working with new parents.” |
| Additionally, it is unclear why Ireland is included and not other countries. | Thank you for highlighting this. A short justification on the Irish context has been added to line 98-100. It reads: ”The Irish context is particularly relevant given the short paternity leave provision and gendered caregiving norms that may shape how sleep loss and early parenting are negotiated.” |
| Several references in this section are quite old; I suggest updating the literature review. | The older papers have been removed. |
| METHODS  This section needs more information.  In the design, you only state the instrument used (interviews). What type of study is this? Which research tradition does it follow? | Thank you for these comments. The type of study and research tradition has been made explicit in line 106. It now reads “This study employed a qualitative research design using semi-structured one-to-one interviews with first-time parents to explore their experiences and perceptions of sleep loss, tiredness and wellbeing during the postpartum period. One-to-one interviews were appropriate as they allow for an understanding of unique and personal experiences, rather than group norms or patterns.^21,22^ This study was underpinned by a constructionist epistemology, recognising that experiences of sleep loss and wellbeing are socially shaped and interpreted within broader cultural and gendered contexts.” |
| Did you use a reporting guideline? | Clear reference to the Standards for Reporting Qualitative Research (SRQR) has been added in line 112-113. It states “This study is reported in line with the Standards for Reporting Qualitative Research.” |
| Please also include the exclusion criteria of the participants. | The exclusion criteria has been added to line 104-106. The new text reads “Exclusion criteria consisted of those under the age of eighteen, who did not have sufficient English language competencies, and experienced a complicated vaginal birth or emergency caesarean section. “ |
| Can you provide the characteristics of the interviewer? In qualitative research, these are important. | Thank you for highlighting this, Further characteristics have been added to line 143-145. It now reads “All interviews were conducted by the lead author, a PhD candidate with a background in sociology and qualitative research. She had no prior relationship with participants and adopted a reflexive approach throughout the data collection and analysis.” |
| How did you decide when to stop the interviews? Did you consider data saturation? | We appreciate your comment. This has now been made clear in line 140-143. It now says “The decision to cease recruitment was informed by the developing analysis, with similar patterns being observed across the participants’ accounts. Rather than seeking data saturation, the study prioritised a depth of rich data to address the research aims.” |
| RESULTS  This section is well written and informative. No comments. | Thank you for this feedback. |
| DISCUSSION  The discussion is informative.  However, the importance of this study for healthcare workers is not clear. | A paragraph on the relevance to healthcare workers has been added into line 405-409. It reads: “Our findings have important implications for clinical practices. As nurses and midwives are well positioned to recognise the psychological and physical dimensions of postpartum sleep loss, routine discussions of sleep should be incorporated into postnatal care. This should include addressing gendered divisions of care and offering practical strategies for managing sleep in the early postpartum period.” |
| The implications of the study at the clinical, managerial, educational, and research levels are not described. | This comment has been addressed in line 405-410. It now reads “Our findings have important implications for clinical practices. As nurses and midwives are well positioned to recognise the psychological and physical dimensions of postpartum sleep loss, routine discussions of sleep should be incorporated into postnatal care. This should include addressing gendered divisions of care and offering practical strategies for managing sleep in the early postpartum period. Furthermore, future research should take into consideration the gendered nature of sleep loss when examining first-time parents’ experiences of sleep.” |
| Finally, I suggest including methodological limitations, not just those related to the participants. | Thank you for highlighting this. A sentence has been added to line 411. It now reads “There were several limitations to our study. As the study aimed to explore lived experiences, generalisability may not be applicable.” |
| **Reviewer 2** |  |
| Adding the “six-months” period to the objective in the abstract would improve clarity. For example: “We examined first-time parents' perceptions, experiences and attitudes to sleep loss, tiredness and wellbeing during the first six months postpartum in Ireland.” | Thank you for your comment. This has been adjusted on line 25. |
| Lines 37–38 of the abstract (currently in the Conclusions section) would fit better within the Results. Consequently, I would suggest revising the Conclusions to focus more explicitly on implications for practice, in addition to those for future research. | Thank you kindly for this suggestion. The abstract conclusion has been updated to better reflect this in line 41-44. It now reads: “These findings highlight the need for healthcare professionals to recognise postpartum sleep loss as both a physiological and psychological experience, and to incorporate holistic, routine discussions of sleep into postnatal care.” |
| The number of parents recruited should be clearly reported in the Results section. The same applies to the abstract and to the “Participants” subsection in the Methods (line 97). Similarly, the sentence “There were three couples within the sample group, resulting in fewer infants than parents (infants n = 11)” should be relocated to the Results. | The sentence “There were three couples within the sample group, resulting in less infants than parents (infants n=11).” was added under the findings section in line 146-147. |
| Exclusion criteria should also be explicitly listed under the “Participants” subsection. | The exclusion criteria has been added to line 104-106. The new text reads “Exclusion criteria consisted of those under the age of eighteen, who did not have sufficient English language competencies, and experienced a complicated vaginal birth or emergency caesarean section. “ |
| Lines 108–110: a reference to the sources from which the interview guide was developed should be provided. | A reference to previous study used has been added to line 131. |
| In addition to describing the qualitative data analysis, the manuscript should include a brief explanation of how other collected data (e.g., demographic variables) were analyzed. For instance, specify whether descriptive statistics were used (percentages, frequencies) and clarify how continuous variables such as age were reported (mean and standard deviation or median and interquartile range). These data should also be presented consistently in the first paragraph of the Results. | Thank you for the comment. This has been addressed in line 162-163. It now reads “Descriptive statistics were used to examine the demographic state, with ages being reported with a mean and standard deviation.” |
| The participant demographics table should be revised to ensure anonymity, avoiding the use of names. For example, labels such as “Couple 1,” “Couple 2,” or “Interview 1” could be used. | Thank you for this comment. We value participants' right to confidentiality and so any names that appear are pseudonyms. This has been made clearer in the participant table. |
| Line 148: given the qualitative nature of the study, it would be more appropriate to use the term “Findings” instead of “Results.” For example: “The findings are presented under…”. Please also clearly describe the subthemes identified (e.g., “Paternity leave,” “Mother’s work after childbirth”), and consider numbering subheadings to improve readability. | These suggestions have been implemented throughout the manuscript.   - Changing results to findings - Numbering headings and subheadings - Briefly defining each subheading where it was previously unexplained |
| Instead of reporting participants’ names alongside quotations (e.g., “(MAX)”), use anonymized identifiers such as “(Interview 1).” | Thank you for this comment. We value participants' right to confidentiality and so any names that appear are pseudonyms. |
| In the Conclusions, it would strengthen the manuscript to include implications for clinical practice, particularly for nurses and midwives, as well as potential implications for education. | Thank you for this suggestion to improve the manuscript. The conclusion has been extended to include such suggestions, from line 394-403. The new text reads “Preparing healthcare professionals to engage with postpartum sleep may enhance the support provided to new parents. Antenatal and postnatal education programmes should better equip parents by setting realistic expectations around sleep loss and tiredness, and encourage shared caregiving practices and provide coping strategies that acknowledge the emotional and relation challenges that can arise during the postpartum period.” |
