# Supplementary material for: “Tired but wired” - First-time parents lived experiences of sleep loss, tiredness and wellbeing during the first six months postpartum
Source: PLoS One. 2026 Oct 27;21(10):e0347558. doi: 10.1371/journal.pone.0347558 (PMC13712679; doi:10.1371/journal.pone.0347558)
Supplement: Peer review history

# Peer review: Decision Letter 0

**DOI:** 10.1371/journal.pone.0347558.r001

**Authors:** Laura Brunelli

30 Apr 2026

Dear Dr. Hughes Spence,

Thank you for submitting your manuscript to PLOS ONE. After careful consideration, we feel that it has merit but does not fully meet PLOS ONE’s publication criteria as it currently stands. Therefore, we invite you to submit a revised version of the manuscript that addresses the points raised during the review process.

==============================

**ACADEMIC EDITOR: Please address the reviewers' feedbacks to improve your work.**

==============================

Please submit your revised manuscript by Jun 14 2026 11:59PM. If you will need more time than this to complete your revisions, please reply to this message or contact the journal office at plosone@plos.org. When you're ready to submit your revision, log on to https://www.editorialmanager.com/pone/ and select the 'Submissions Needing Revision' folder to locate your manuscript file.

- A letter that responds to each point raised by the academic editor and reviewer(s). You should upload this letter as a separate file labeled 'Response to Reviewers'.
- A marked-up copy of your manuscript that highlights changes made to the original version. You should upload this as a separate file labeled 'Revised Manuscript with Track Changes'.
- An unmarked version of your revised paper without tracked changes. You should upload this as a separate file labeled 'Manuscript'.

If you would like to make changes to your financial disclosure, please include your updated statement in your cover letter. Guidelines for resubmitting your figure files are available below the reviewer comments at the end of this letter.

If applicable, we recommend that you deposit your laboratory protocols in protocols.io to enhance the reproducibility of your results. Protocols.io assigns your protocol its own identifier (DOI) so that it can be cited independently in the future. For instructions see: https://journals.plos.org/plosone/s/submission-guidelines#loc-laboratory-protocols. Additionally, PLOS ONE offers an option for publishing peer-reviewed Lab Protocol articles, which describe protocols hosted on protocols.io. Read more information on sharing protocols at https://plos.org/protocols?utm_medium=editorial-email&utm_source=authorletters&utm_campaign=protocols.

As the corresponding author, your ORCID iD is verified in the submission system and will appear in the published article. PLOS supports the use of ORCID, and we encourage all coauthors to register for an ORCID iD and use it as well. Please encourage your coauthors to verify their ORCID iD within the submission system before final acceptance, as unverified ORCID iDs will not appear in the published article. *Only*  the individual author can complete the verification step; PLOS staff *cannot*  verify ORCID iDs on behalf of authors.

We look forward to receiving your revised manuscript.

Kind regards,

Laura Brunelli, MD, PhD

Academic Editor

PLOS One

Journal requirements:

When submitting your revision, we need you to address these additional requirements.

1. Please ensure that your manuscript meets PLOS ONE's style requirements, including those for file naming. The PLOS ONE style templates can be found at

https://journals.plos.org/plosone/s/file?id=wjVg/PLOSOne_formatting_sample_main_body.pdf and

https://journals.plos.org/plosone/s/file?id=ba62/PLOSOne_formatting_sample_title_authors_affiliations.pdf

2. Peer review at PLOS  One is not double-blinded (https://journals.plos.org/plosone/s/editorial-and-peer-review-process). For this reason, authors should include in the revised manuscript all the information removed for blind review.

3. In the online submission form, you indicated that [Insert text from online submission form here].

All PLOS journals now require all data underlying the findings described in their manuscript to be freely available to other researchers, either 1. In a public repository, 2. Within the manuscript itself, or 3. Uploaded as supplementary information.

This policy applies to all data except where public deposition would breach compliance with the protocol approved by your research ethics board. If your data cannot be made publicly available for ethical or legal reasons (e.g., public availability would compromise patient privacy), please explain your reasons on resubmission and your exemption request will be escalated for approval.

4. We note that this data set consists of interview transcripts. Can you please confirm that all participants gave consent for interview transcript to be published?

If they DID provide consent for these transcripts to be published, please also confirm that the transcripts do not contain any potentially identifying information (or let us know if the participants consented to having their personal details published and made publicly available). We consider the following details to be identifying information:

- Names, nicknames, and initials

- Age more specific than round numbers

- GPS coordinates, physical addresses, IP addresses, email addresses

- Information in small sample sizes (e.g. 40 students from X class in X year at X university)

- Specific dates (e.g. visit dates, interview dates)

- ID numbers

Or, if the participants DID NOT provide consent for these transcripts to be published:

- Provide a de-identified version of the data or excerpts of interview responses

- Provide information regarding how these transcripts can be accessed by researchers who meet the criteria for access to confidential data, including:

a) the grounds for restriction

b) the name of the ethics committee, Institutional Review Board, or third-party organization that is imposing sharing restrictions on the data

c) a non-author, institutional point of contact that is able to field data access queries, in the interest of maintaining long-term data accessibility.

d) Any relevant data set names, URLs, DOIs, etc. that an independent researcher would need in order to request your minimal data set.

For further information on sharing data that contains sensitive participant information, please see: https://journals.plos.org/plosone/s/data-availability#loc-human-research-participant-data-and-other-sensitive-data

If there are ethical, legal, or third-party restrictions upon your dataset, you must provide all of the following details (https://journals.plos.org/plosone/s/data-availability#loc-acceptable-data-access-restrictions):

1. A complete description of the dataset

2. The nature of the restrictions upon the data (ethical, legal, or owned by a third party) and the reasoning behind them

3. The full name of the body imposing the restrictions upon your dataset (ethics committee, institution, data access committee, etc)

4. If the data are owned by a third party, confirmation of whether the authors received any special privileges in accessing the data that other researchers would not have

5. Direct, non-author contact information (preferably email) for the body imposing the restrictions upon the data, to which data access requests can be sent

If the reviewer comments include a recommendation to cite specific previously published works, please review and evaluate these publications to determine whether they are relevant and should be cited. There is no requirement to cite these works unless the editor has indicated otherwise.

[Note: HTML markup is below. Please do not edit.]

Reviewers' comments:

Reviewer's Responses to Questions

**Comments to the Author**

1. Is the manuscript technically sound, and do the data support the conclusions?

Reviewer #1: Partly

Reviewer #2: Yes

**********

2. Has the statistical analysis been performed appropriately and rigorously?

Reviewer #1: N/A

Reviewer #2: No

**********

3. Have the authors made all data underlying the findings in their manuscript fully available?

The PLOS Data policy

Reviewer #1: Yes

Reviewer #2: Yes

**********

4. Is the manuscript presented in an intelligible fashion and written in standard English?

Reviewer #1: Yes

Reviewer #2: No

**********

Reviewer #1: Dear authors,

Thank you for your interesting paper.

Please find below some suggestions to improve it.

ABSTRACT

This section is well structured and informative. No comments.

INTRODUCTION

The introduction is well structured.

However, it is not clear what the scientific gap in the literature is and how this manuscript contributes to clinical practice.

Additionally, it is unclear why Ireland is included and not other countries.

Several references in this section are quite old; I suggest updating the literature review.

METHODS

This section needs more information.

In the design, you only state the instrument used (interviews). What type of study is this? Which research tradition does it follow?

Did you use a reporting guideline?

Please also include the exclusion criteria of the participants.

Can you provide the characteristics of the interviewer? In qualitative research, these are important.

How did you decide when to stop the interviews? Did you consider data saturation?

RESULTS

This section is well written and informative. No comments.

DISCUSSION

The discussion is informative.

However, the importance of this study for healthcare workers is not clear.

The implications of the study at the clinical, managerial, educational, and research levels are not described.

Finally, I suggest including methodological limitations, not just those related to the participants.

Reviewer #2: This paper on parents’ sleep loss during the first six months postpartum is both interesting and contributes novel insights to the existing literature.

Adding the “six-months” period to the objective in the abstract would improve clarity. For example: “We examined first-time parents' perceptions, experiences and attitudes to sleep loss, tiredness and wellbeing during the first six months postpartum in Ireland.”

Lines 37–38 of the abstract (currently in the Conclusions section) would fit better within the Results. Consequently, I would suggest revising the Conclusions to focus more explicitly on implications for practice, in addition to those for future research.

The number of parents recruited should be clearly reported in the Results section. The same applies to the abstract and to the “Participants” subsection in the Methods (line 97). Similarly, the sentence “There were three couples within the sample group, resulting in fewer infants than parents (infants n = 11)” should be relocated to the Results.

Exclusion criteria should also be explicitly listed under the “Participants” subsection.

Lines 108–110: a reference to the sources from which the interview guide was developed should be provided.

In addition to describing the qualitative data analysis, the manuscript should include a brief explanation of how other collected data (e.g., demographic variables) were analyzed. For instance, specify whether descriptive statistics were used (percentages, frequencies) and clarify how continuous variables such as age were reported (mean and standard deviation or median and interquartile range). These data should also be presented consistently in the first paragraph of the Results.

The participant demographics table should be revised to ensure anonymity, avoiding the use of names. For example, labels such as “Couple 1,” “Couple 2,” or “Interview 1” could be used.

Line 148: given the qualitative nature of the study, it would be more appropriate to use the term “Findings” instead of “Results.” For example: “The findings are presented under…”. Please also clearly describe the subthemes identified (e.g., “Paternity leave,” “Mother’s work after childbirth”), and consider numbering subheadings to improve readability.

Instead of reporting participants’ names alongside quotations (e.g., “(MAX)”), use anonymized identifiers such as “(Interview 1).”

In the Conclusions, it would strengthen the manuscript to include implications for clinical practice, particularly for nurses and midwives, as well as potential implications for education.

**********

what does this mean?). If published, this will include your full peer review and any attached files.

If you choose “no”, your identity will remain anonymous but your review may still be made public.

**Do you want your identity to be public for this peer review?**  For information about this choice, including consent withdrawal, please see our Privacy Policy

Reviewer #1: No

Reviewer #2: No

**********

[NOTE: If reviewer comments were submitted as an attachment file, they will be attached to this email and accessible via the submission site. Please log into your account, locate the manuscript record, and check for the action link "View Attachments". If this link does not appear, there are no attachment files.]

To ensure your figures meet our technical requirements, please review our figure guidelines: https://journals.plos.org/plosone/s/figures

You may also use PLOS’s free figure tool, NAAS, to help you prepare publication quality figures: https://journals.plos.org/plosone/s/figures#loc-tools-for-figure-preparation.

NAAS will assess whether your figures meet our technical requirements by comparing each figure against our figure specifications.

# Author comment: Author response to Decision Letter 1

**DOI:** 10.1371/journal.pone.0347558.r002

29 May 2026

Thank you to the reviews for their thoughtful and insightful comments. The feedback is greatly appreciated and each has been addressed in the Response to Reviews letter that is attached with this revision.

# Editor report: Decision Letter 1

**DOI:** 10.1371/journal.pone.0347558.r003

**Authors:** Laura Brunelli

12 Jun 2026

Dear Dr. Hughes Spence,

Thank you for submitting your manuscript to PLOS ONE. After careful consideration, we feel that it has merit but does not fully meet PLOS ONE’s publication criteria as it currently stands. Therefore, we invite you to submit a revised version of the manuscript that addresses the points raised during the review process.

Please submit your revised manuscript by Jul 27 2026 11:59PM. If you will need more time than this to complete your revisions, please reply to this message or contact the journal office at plosone@plos.org. When you're ready to submit your revision, log on to https://www.editorialmanager.com/pone/ and select the 'Submissions Needing Revision' folder to locate your manuscript file.

- A letter that responds to each point raised by the academic editor and reviewer(s). You should upload this letter as a separate file labeled 'Response to Reviewers'.
- A marked-up copy of your manuscript that highlights changes made to the original version. You should upload this as a separate file labeled 'Revised Manuscript with Track Changes'.
- An unmarked version of your revised paper without tracked changes. You should upload this as a separate file labeled 'Manuscript'.

If you would like to make changes to your financial disclosure, please include your updated statement in your cover letter. Guidelines for resubmitting your figure files are available below the reviewer comments at the end of this letter.

If applicable, we recommend that you deposit your laboratory protocols in protocols.io to enhance the reproducibility of your results. Protocols.io assigns your protocol its own identifier (DOI) so that it can be cited independently in the future. For instructions see: https://journals.plos.org/plosone/s/submission-guidelines#loc-laboratory-protocols. Additionally, PLOS ONE offers an option for publishing peer-reviewed Lab Protocol articles, which describe protocols hosted on protocols.io. Read more information on sharing protocols at https://plos.org/protocols?utm_medium=editorial-email&utm_source=authorletters&utm_campaign=protocols.

As the corresponding author, your ORCID iD is verified in the submission system and will appear in the published article. PLOS supports the use of ORCID, and we encourage all coauthors to register for an ORCID iD and use it as well. Please encourage your coauthors to verify their ORCID iD within the submission system before final acceptance, as unverified ORCID iDs will not appear in the published article. *Only*  the individual author can complete the verification step; PLOS staff *cannot*  verify ORCID iDs on behalf of authors.

We look forward to receiving your revised manuscript.

Kind regards,

Laura Brunelli, MD, PhD

Academic Editor

PLOS One

Journal Requirements:

If the reviewer comments include a recommendation to cite specific previously published works, please review and evaluate these publications to determine whether they are relevant and should be cited. There is no requirement to cite these works unless the editor has indicated otherwise.

Please review your reference list to ensure that it is complete and correct. If you have cited papers that have been retracted, please include the rationale for doing so in the manuscript text, or remove these references and replace them with relevant current references. Any changes to the reference list should be mentioned in the rebuttal letter that accompanies your revised manuscript. If you need to cite a retracted article, indicate the article’s retracted status in the References list and also include a citation and full reference for the retraction notice.

Additional Editor Comments:

Dear Authors,

Thank you for revising your work.

I appreciate that you have addressed all reviewers' feedback, and I think the paper is much clearer now.

As you have justified the inclusion of Irish participants based on the short paternity leave provided, please discuss this aspect further in the discussion section. You may add a paragraph comparing and contrasting the Irish situation with other European or high-income countries regarding the duration of paternity leave and/or the distribution of leave between mother and father. This would enhance interest among international readers and improve the paper's relevance at the international level.

Kind regards

[Note: HTML markup is below. Please do not edit.]

[NOTE: If reviewer comments were submitted as an attachment file, they will be attached to this email and accessible via the submission site. Please log into your account, locate the manuscript record, and check for the action link "View Attachments". If this link does not appear, there are no attachment files.]

To ensure your figures meet our technical requirements, please review our figure guidelines: https://journals.plos.org/plosone/s/figures

You may also use PLOS’s free figure tool, NAAS, to help you prepare publication quality figures: https://journals.plos.org/plosone/s/figures#loc-tools-for-figure-preparation.

NAAS will assess whether your figures meet our technical requirements by comparing each figure against our figure specifications.

# Author comment: Author response to Decision Letter 2

**DOI:** 10.1371/journal.pone.0347558.r004

22 Jun 2026

Thank you for the comment and suggestion for improving the manuscript. This suggestion has been implemented and inserted into the manuscript, from line 366 to 380. It reads:

“However, the structural policies that shape this unequitable distribution of labour must also be acknowledged. Ireland’s state paid paternity leave is the minimum requirement under European Union laws and legislation30. The state payment for paternity leave is currently €299 per week, with economic loss being a possible factor to the low uptake of paternity leave. Internationally, the average paid paternity leave amongst members of the Organisation for Economic Co-operation and Development (OECD) is 2.4 weeks,31 with Spain offering the longest period of paid paternity leave at 16 weeks. However, amongst OECD countries, fathers can avail of paid leave of 8.1weeks while retaining their full earnings, with Japan offering 31.1 weeks at full earning31. The Irish state’s lack of financial support for new fathers reinforces a culture where women in Ireland still carry out the majority of care during the postpartum period, while fathers miss out on the early months of parenthood due to financial necessity. In line with other countries, Irish policy should consider extending the duration and payment of paid paternity leave beyond the minimum statutory requirements. Evidence suggests that well-compensated paternity policies are associated with higher uptake amongst new fathers and greater gender equality31, and potentially, increased opportunities for rest and wellbeing during the postpartum period. “

Attachment Submitted filename: LASTResponse to reviewers.docx

# Peer review: Decision Letter 2

**DOI:** 10.1371/journal.pone.0347558.r005

**Authors:** Kshitij Karki

15 Sep 2026

Dear Dr. Hughes Spence,

Please submit your revised manuscript by Oct 30 2026 11:59PM, you will need more time than this to complete your revisions, please reply to this message or contact the journal office at plosone@plos.org. When you're ready to submit your revision, log on to https://www.editorialmanager.com/pone/ and select the 'Submissions Needing Revision' folder to locate your manuscript file.

- A letter that responds to each point raised by the academic editor and reviewer(s). You should upload this letter as a separate file labeled 'Response to Reviewers'.
- A marked-up copy of your manuscript that highlights changes made to the original version. You should upload this as a separate file labeled 'Revised Manuscript with Track Changes'.
- An unmarked version of your revised paper without tracked changes. You should upload this as a separate file labeled 'Manuscript'.

If you would like to make changes to your financial disclosure, please include your updated statement in your cover letter. Guidelines for resubmitting your figure files are available below the reviewer comments at the end of this letter.

If applicable, we recommend that you deposit your laboratory protocols in protocols.io to enhance the reproducibility of your results. Protocols.io assigns your protocol its own identifier (DOI) so that it can be cited independently in the future. For instructions see: https://journals.plos.org/plosone/s/submission-guidelines#loc-laboratory-protocols. Additionally, PLOS One offers an option for publishing peer-reviewed Lab Protocol articles, which describe protocols hosted on protocols.io. Read more information on sharing protocols at https://plos.org/protocols?utm_medium=editorial-email&utm_source=authorletters&utm_campaign=protocols.

As the corresponding author, your ORCID iD is verified in the submission system and will appear in the published article. PLOS supports the use of ORCID, and we encourage all coauthors to register for an ORCID iD and use it as well. Please encourage your coauthors to verify their ORCID iD within the submission system before final acceptance, as unverified ORCID iDs will not appear in the published article. *Only*  the individual author can complete the verification step; PLOS staff *cannot*  verify ORCID iDs on behalf of authors.

We look forward to receiving your revised manuscript.

Kind regards,

Kshitij Karki, MPH, MA

Academic Editor

PLOS One

Journal Requirements:

If the reviewer comments include a recommendation to cite specific previously published works, please review and evaluate these publications to determine whether they are relevant and should be cited. There is no requirement to cite these works unless the editor has indicated otherwise.

Please review your reference list to ensure that it is complete and correct. If you have cited papers that have been retracted, please include the rationale for doing so in the manuscript text, or remove these references and replace them with relevant current references. Any changes to the reference list should be mentioned in the rebuttal letter that accompanies your revised manuscript. If you need to cite a retracted article, indicate the article’s retracted status in the References list and also include a citation and full reference for the retraction notice.

[Note: HTML markup is below. Please do not edit.]

Reviewers' comments:

Reviewer's Responses to Questions

**Comments to the Author**

Reviewer #3: All comments have been addressed

Reviewer #4: (No Response)

**********

2. Is the manuscript technically sound, and do the data support the conclusions?

Reviewer #3: Yes

Reviewer #4: Yes

**********

3. Has the statistical analysis been performed appropriately and rigorously?

Reviewer #3: N/A

Reviewer #4: N/A

**********

4. Have the authors made all data underlying the findings in their manuscript fully available?

The PLOS Data policy

Reviewer #3: No

Reviewer #4: Yes

**********

5. Is the manuscript presented in an intelligible fashion and written in standard English?

Reviewer #3: Yes

Reviewer #4: Yes

**********

Reviewer #3: The authors performed a study titled "Tired but wired" - First-time parents' lived experiences of sleep loss, tiredness and wellbeing during the first six months postpartum.

The manuscript is methodologically consistent with the reflexive thematic analysis (RTA) tradition. It declares (Braun & Clarke) and with the Standards for Reporting Qualitative Research. The sample size (n=14) and the decision to prioritise depth over data saturation are appropriate to this method and are already acknowledged as limiting generalizability. My concern is on reporting precision and a small number of framing/transparency issues. Please see my comments here:

1. I observe that claims are reported using unanchored quantifiers — "many female participants," "several participants," "some participants," "commonly," "often" (e.g. lines 196, 257–258, 286, 304, 341). For a reader to be able to judge the weight of each claim, please add the underlying count alongside each qualifier on first use in each theme/subtheme, e.g. "several participants (n=5/14)" or "most mothers (n=8/10)"- which will make existing claims auditable.

2. Participants were recruited via maternity/parenting organizations' mailing lists, social media, and personal-network snowballing. This plausibly selects for parents who are already engaged with parenting/wellbeing discourse (or, conversely, for those experiencing enough difficulty to be motivated to volunteer). I would ask to add a sentence acknowledging this self-selection alongside the existing limitations (gender imbalance, partnered-only sample, no socioeconomic data).

3. The opening empirical claim of the Introduction ("83% of mothers were fatigued between four to six weeks postpartum") rests on a single 2014 study. Please check for more recent prevalence data to anchor the opening claim.

Reviewer #4: Thank you for the opportunity to review your manuscript. I appreciate learning more about this topic, especially from an international perspective (I'm in the US). In addition, I think that paternal wellbeing is an under researched population and you did add to the literature on this topic. I am interested to see what future research you find if you continue digging deeper.

I have a minor recommendation- Line 235, this sentence is missing a word, I think you meant "...certain number of hours of uninterrupted sleep."

Thank you!

**********

what does this mean?). If published, this will include your full peer review and any attached files.

If you choose “no”, your identity will remain anonymous but your review may still be made public.

**Do you want your identity to be public for this peer review?**  For information about this choice, including consent withdrawal, please see our Privacy Policy

Reviewer #3: **Yes:**  Md Aktaruzzaman

Reviewer #4: No

**********

[NOTE: If reviewer comments were submitted as an attachment file, they will be attached to this email and accessible via the submission site. Please log into your account, locate the manuscript record, and check for the action link "View Attachments". If this link does not appear, there are no attachment files.]

To ensure your figures meet our technical requirements, please review our figure guidelines: https://journals.plos.org/plosone/s/figures

You may also use PLOS’s free figure tool, NAAS, to help you prepare publication quality figures: https://journals.plos.org/plosone/s/figures#loc-tools-for-figure-preparation.

NAAS will assess whether your figures meet our technical requirements by comparing each figure against our figure specifications.

# Author comment: Author response to Decision Letter 3

**DOI:** 10.1371/journal.pone.0347558.r006

17 Sep 2026

Responses in document attached

Attachment Submitted filename: LASTSEPTResponse to reviewers.docx

# Peer review: Decision Letter 3

**DOI:** 10.1371/journal.pone.0347558.r007

**Authors:** Kshitij Karki

7 Oct 2026

“Tired but wired” - First-time parents lived experiences of sleep loss, tiredness and wellbeing during the first six months postpartum

PONE-D-26-16303R3

Dear Dr. Shannon Hughes Spence,

We’re pleased to inform you that your manuscript has been judged scientifically suitable for publication and will be formally accepted for publication once it meets all outstanding technical requirements.

Within one week, you’ll receive an e-mail detailing the required amendments. When these have been addressed, you’ll receive a formal acceptance letter and your manuscript will be scheduled for publication.

An invoice will be generated when your article is formally accepted. Please note, if your institution has a publishing partnership with PLOS and your article meets the relevant criteria, all or part of your publication costs will be covered. Please make sure your user information is up-to-date by logging into Editorial Manager at Editorial Manager® and clicking the ‘Update My Information' link at the top of the page. For questions related to billing, please contact billing support.

If your institution or institutions have a press office, please notify them about your upcoming paper to help maximize its impact. If they’ll be preparing press materials, please inform our press team as soon as possible -- no later than 48 hours after receiving the formal acceptance. Your manuscript will remain under strict press embargo until 2 pm Eastern Time on the date of publication. For more information, please contact onepress@plos.org.

Kind regards,

Kshitij Karki, MPH, MA

Academic Editor

PLOS One

Additional Editor Comments (optional):

Thank you for addressing the comments.

Reviewers' comments:

Reviewer's Responses to Questions

**Comments to the Author**

Reviewer #3: All comments have been addressed

Reviewer #4: All comments have been addressed

**********

2. Is the manuscript technically sound, and do the data support the conclusions?

Reviewer #3: Yes

Reviewer #4: Yes

**********

3. Has the statistical analysis been performed appropriately and rigorously?

Reviewer #3: Yes

Reviewer #4: N/A

**********

4. Have the authors made all data underlying the findings in their manuscript fully available?

The PLOS Data policy

Reviewer #3: Yes

Reviewer #4: Yes

**********

5. Is the manuscript presented in an intelligible fashion and written in standard English?

Reviewer #3: Yes

Reviewer #4: Yes

**********

Reviewer #3: I went through the revised version of the manuscript.

The authors have put significant efforts to fix the issues addressed previously, and the quality of presentation has been improved now.

I appreciate the efforts they put on it.

Reviewer #4: Thank you for addressing the grammar concern on line 235. I look forward to reading your future research on paternal wellbeing:)

**********

what does this mean?). If published, this will include your full peer review and any attached files.

If you choose “no”, your identity will remain anonymous but your review may still be made public.

**Do you want your identity to be public for this peer review?**  For information about this choice, including consent withdrawal, please see our Privacy Policy

Reviewer #3: No

Reviewer #4: **Yes:**  Jennifer Clift, PhD, LMSW

**********

# Editor report: Acceptance letter

**DOI:** 10.1371/journal.pone.0347558.r008

**Authors:** Kshitij Karki

PONE-D-26-16303R3

PLOS One

Dear Dr. Hughes Spence,

I'm pleased to inform you that your manuscript has been deemed suitable for publication in PLOS One. Congratulations! Your manuscript is now being handed over to our production team.

At this stage, our production department will prepare your paper for publication. This includes ensuring the following:

* All references, tables, and figures are properly cited

* All relevant supporting information is included in the manuscript submission,

* There are no issues that prevent the paper from being properly typeset

You will receive further instructions from the production team, including instructions on how to review your proof when it is ready. Please keep in mind that we are working through a large volume of accepted articles, so please give us a few days to review your paper and let you know the next and final steps.

Lastly, if your institution or institutions have a press office, please let them know about your upcoming paper now to help maximize its impact. If they'll be preparing press materials, please inform our press team within the next 48 hours. Your manuscript will remain under strict press embargo until 2 pm Eastern Time on the date of publication. For more information, please contact onepress@plos.org.

You will receive an invoice from PLOS for your publication fee after your manuscript has reached the completed accept phase. If you receive an email requesting payment before acceptance or for any other service, this may be a phishing scheme. Learn how to identify phishing emails and protect your accounts at https://explore.plos.org/phishing.

If we can help with anything else, please email us at customercare@plos.org.

Thank you for submitting your work to PLOS One and supporting open access.

Kind regards,

PLOS One Editorial Office Staff

on behalf of

Dr. Kshitij Karki

Academic Editor

PLOS One
